# Pathways to health: Reporting on health co-benefits from urban climate mitigation action varies by sector

**DOI:** 10.1038/s42949-025-00311-y

**Published:** 2025-11-29

**Authors:** Blanca Anton, Andy Haines, Rosemary Green, Nienke Meinsma, Tamzin Reynolds, Sarah Whitmee

**Affiliations:** 1 Department of Population Health, London School of Hygiene & Tropical Medicine, London, UK; 2 Institute of Global Health Innovation, Imperial College London, London, UK

**Keywords:** Climate-change mitigation, Environmental impact

## Abstract

Well-designed city actions to reduce greenhouse gas emissions can also deliver substantial near-term health co-benefits. Improved understanding and reporting of the health benefits from climate mitigation can aid efforts by cities to design and deliver healthy, equitable solutions to the climate crisis. Using global data from the 2022 CDP-ICLEI Track cities questionnaire, we analysed factors that may influence the awareness and identification of health co-benefits from climate mitigation. Actions from the transport and AFOLU sector were five to eight times more likely to report health co-benefits than other sectors, regardless of which region the action was undertaken. There was no significant difference between actions in the pre-implementation stage compared to actions that were underway. The findings highlight the need to raise awareness about the potential health benefits linked to climate mitigation among urban policymakers across all sectors to help deliver an equitable transition to a healthy, net zero future.

## Introduction

Cities play a vital role in the transition to a healthy, net-zero future. Over half of the world’s population currently reside in urban settings and more than 1 million people per week move to cities^1^. Most urban population growth is projected to take place in low-and middle-income countries (LMIC) over coming years, largely in Asia and Africa. As a major source of national GHG emissions, cities play an important role in delivering agreed national mitigation targets under the Paris Agreement. Cities are hotspots for human activity and infrastructure and thus account for approximately 70% of global greenhouse gas (GHG) emissions, posing a major opportunity for emission reductions^1^ through improved urban planning and design^2^.

Local governments are increasingly leading the way with innovative climate action and policies, particularly in sectors such as transportation, waste management, buildings, and construction^3^. Several city-level initiatives have emerged to foster collaboration among cities committed to climate action including CDP Cities (formerly the Carbon Disclosure Project), ICLEI (Local Governments for Sustainability), C40 Cities, and the Resilient Cities Network (formerly 100 Resilient Cities). These initiatives aim to share best practices, set ambitious goals, and advocate for policies that address climate change. By connecting like-minded cities, they enhance the capacity for local governments to implement effective climate actions and policies, ultimately contributing to global sustainability efforts. CDP Cities, in conjunction with ICLEI developed the CDP-ICLEI Track, the world’s leading climate reporting platform for local governments to report their environmental impact data on an annual basis. CDP began collecting data from cities in 2010, assisting them in systematically tracking their climate actions and impacts^4^.

Many cities are increasingly facing other compounding pressures and challenges including increasing unemployment, lack of affordable housing, and over-stretched infrastructure, with cascading impacts for the health and financial security of their inhabitants^5,6^. City leaders facing multiple pressures may, therefore, struggle to prioritise climate mitigation action, and despite the urgent need for change, cities often face major barriers to implementing climate action^6^. Over the last few decades, numerous efforts have been undertaken to integrate health into the climate agenda. One policy lever with which to accelerate action on urban mitigation is to communicate and capitalise on the delivery of near-term health co-benefits, which could act as a pivotal factor in facilitating cities to align climate action with other developmental needs, serving as a dual solution^7^.

The term ‘*co-benefits*’ has been defined in various ways^8^. Here, we use the Intergovernmental Panel on Climate Change (IPCC) definition of co-benefits, or so-called ‘ancillary gains’, as secondary and side gains from mitigation policies primarily directed at emission reductions^9^. These gains can encompass economic, health, social, or environmental benefits. Such ancillary gains are often interlinked: social co-benefits such as reduced fuel poverty or economic co-benefits, for example job creation, can also have implications for health. Health co-benefits from climate mitigation action in cities can be realised through well-evidenced pathways including reductions in air pollution from replacing fossil fuels with clean, renewable energy, promotion of active travel, i.e., walking and cycling, and increased consumption of healthy and sustainable diets. Physical and mental health co-benefits can also result from nature-based solutions, including from exposure to nature and the provision of ecosystem services^7^.

The potential magnitude of health co-benefits from climate action in the urban environment is substantial, and there has been an increase in literature modelling, quantifying, and assessing the impacts of mitigation actions on health^7^. A recent national level modelling study showed that the health co-benefits of delivering actions in line with Nationally Determined Contributions (NDCs) from just nine countries (Brazil, China, Germany, India, Indonesia, Nigeria, South Africa, the UK, and the USA) could result in an estimated annual reduction of 1.18 million air pollution-related premature deaths, 5.86 million diet-related deaths and 1.15 million deaths due to physical inactivity by 2040^10^. The extent to which this research has influenced wider political and public engagement has received limited attention and barriers, such as structural challenges, vested interests, and discourse focusing on economic approaches remain that hinder the inclusion and consideration of health impacts in climate change policymaking^11^

Communicating the co-benefits of climate action can help overcome barriers, for example, policymakers often rely on public support to successfully implement policies, and current evidence suggests that the public is more likely to support climate actions if the co-benefits of those actions are emphasised^12^. Economic barriers also exist, with short-term costs and benefits outweighing the long-term benefits from climate action in decision-making processes^13^. As co-benefits are often realised in the near-term and the health and welfare effects are experienced directly by those bearing the costs for action, including taxpayers or consumers, this can help build support for change. For example, the health impacts of mitigation actions that reduce air pollution are directly experienced by local citizens in their daily lives through decreased respiratory and irritation symptoms^12,14,15^. Examples from China and the United States, where mitigation efforts were pursued through highlighting the health benefits from reduced air pollution, suggest that health co-benefits can drive mitigation policies^11^.

To address barriers and better inform future efforts to target capacity support and research in urban environments on the health co-benefits from climate mitigation action, we tested whether the reporting of health co-benefits is associated with characteristics that can plausibly be linked to awareness, implementation and communication of climate action and its benefits at the city level. Existing published evidence on health co-benefits of climate action (both from modelling studies and evaluated interventions) is strongly skewed towards originating from high-income countries (HIC) and the number of publications from HIC, especially from North America, Europe, and Oceania, is currently estimated to be almost ten times greater than the number of publications from low-income countries^16,17^. We therefore hypothesize that mitigation actions from HIC and selected regions would be more likely to report health co-benefits due to better communication and evidence from these regions. Current reviews also show particularly strong sectoral evidence base on co-benefits from climate mitigation actions in the transport, waste, and energy sectors^8^. We therefore expect that the health co-benefits of these sectors might be more commonly reported and understood in comparison to other sectors. In addition, most case studies on urban climate actions are from large cities (over 1 million inhabitants) and from selected mega-cities (over 10 million inhabitants)^17^. Larger cities typically have more resources and capacity^18^, we therefore expect a positive association between the reporting of health co-benefits and city population size. We also tested if the likelihood for reporting health co-benefits varies by implementation status as it may be plausibly expected that due to a lack of health impact assessments of implemented mitigation actions, health co-benefits are more commonly reported pre-operation. Finally, given economic concerns often outweigh other potential benefits in resource constrained cities we tested whether reporting of health co-benefits is significantly more likely compared to other kinds of ancillary benefits thought to be linked to climate action, namely economic (e.g., job creation, revenue generation, increased labour conditions), environmental (e.g., improved water/ soil quality, protected/ improved biodiversity and ecosystem services) and social co-benefits (e.g. improved mobility and access, increased social inclusion, equality and justice, improved education and public awareness on climate issues).

We used data on health co-benefits and climate mitigation actions reported by cities in response to the 2022 CDP-ICLEI Track questionnaire round, which was published in 2023. To our best knowledge, no analysis on the reported health co-benefits using the CDP dataset has been conducted to date. This paper fills an important research gap to better understand factors determining whether cities report on the health co-benefits of climate mitigation actions.

## Results

### Global reporting of urban climate mitigation actions

A total of 792 cities reported 5040 GHG mitigation actions to CDP in 2022. After removing missing data on sector and implementation status, we conducted the analysis with 584 cities and 3835 reported actions (Table 1). Most of the actions were from cities in North America (1322, 34.5%) and Europe (1050, 27.4%), whereas cities in South Asia (79, 2.1%), Africa (73, 1.9%), and the Middle East (43, 1.1%) reported the fewest actions. Most actions, 2753 (71.6%) were reported by cities that have less than 1 million inhabitants (446 cities), whereas 127 large cities (over 1 million inhabitants) reported 1043 (27.1%) actions and 11 megacities (over 10 million inhabitants) reported 39 (1%) of mitigation actions.

**Table 1:** Data characteristics

Variable	*N* (%)
Cities	584 (100)
Mitigation actions	3835 (100)
CDP region	
North America	1322 (34.5)
Europe	1050 (27.4)
Latin America	781 (20.4)
East Asia	197 (5.1)
Southeast Asia	166 (4.3)
South Asia	79 (2.1)
Oceania	124 (3.2)
Africa	73 (1.9)
Middle East	43 (1.1)
**Sector**	
Energy	1661 (43.3)
Transportation	1154 (30.1)
Waste	636 (16.6)
AFOLU	326 (8.5)
Industry	58 (1.5)
**Implementation Status**	
In Operation	1285 (33.5)
Pre-operation	2550 (66.5)
**Population**	
> 10 million	39 (1)
1 million–10 million	1043 (27.2)
< 1 million	2753 (71.8)
**Co-benefits**^**a**^	
Economic	2328 (34.6)
Social	2037 (30.2)
Health	1488 (22.1)
Environment	884 (13.1)
**Health co-benefits**	
Improved air quality^b^	972 (36.8)
Improved mental wellbeing/ quality of life	346 (13.1)
Improved physical health	309 (11.7)
Improved road safety^c^	232 (8.8)
Improved preparedness for health service delivery	239 (9.1)
Reduced health costs	172 (6.5)
Reduced health impacts from extreme heat or cold weather	125 (4.7)
Reduced premature deaths	83 (3.1)
Increased food security^c^	72 (2.7)
Reduced disaster/disease/contamination-related health impacts	67 (2.5)
Improved public health	21 (0.8)

^a^Numbers of co-benefits do not add up to 3835 as one mitigation action can have multiple co-benefits. ^b^We used the health co-benefits categories provided by the 2022 CDP Cities questionnaire, however, we define ‘Improved air quality’ as an exposure and a risk factor for a range of health outcomes. ^c^‘Increased food security’ and ‘Improved road safety’ are categorised as social co-benefits in the 2022 CDP Cities questionnaire but we recoded these as health co-benefits. More information on the variables is available in the methods section and in supplementary information.

**Fig. 1:**
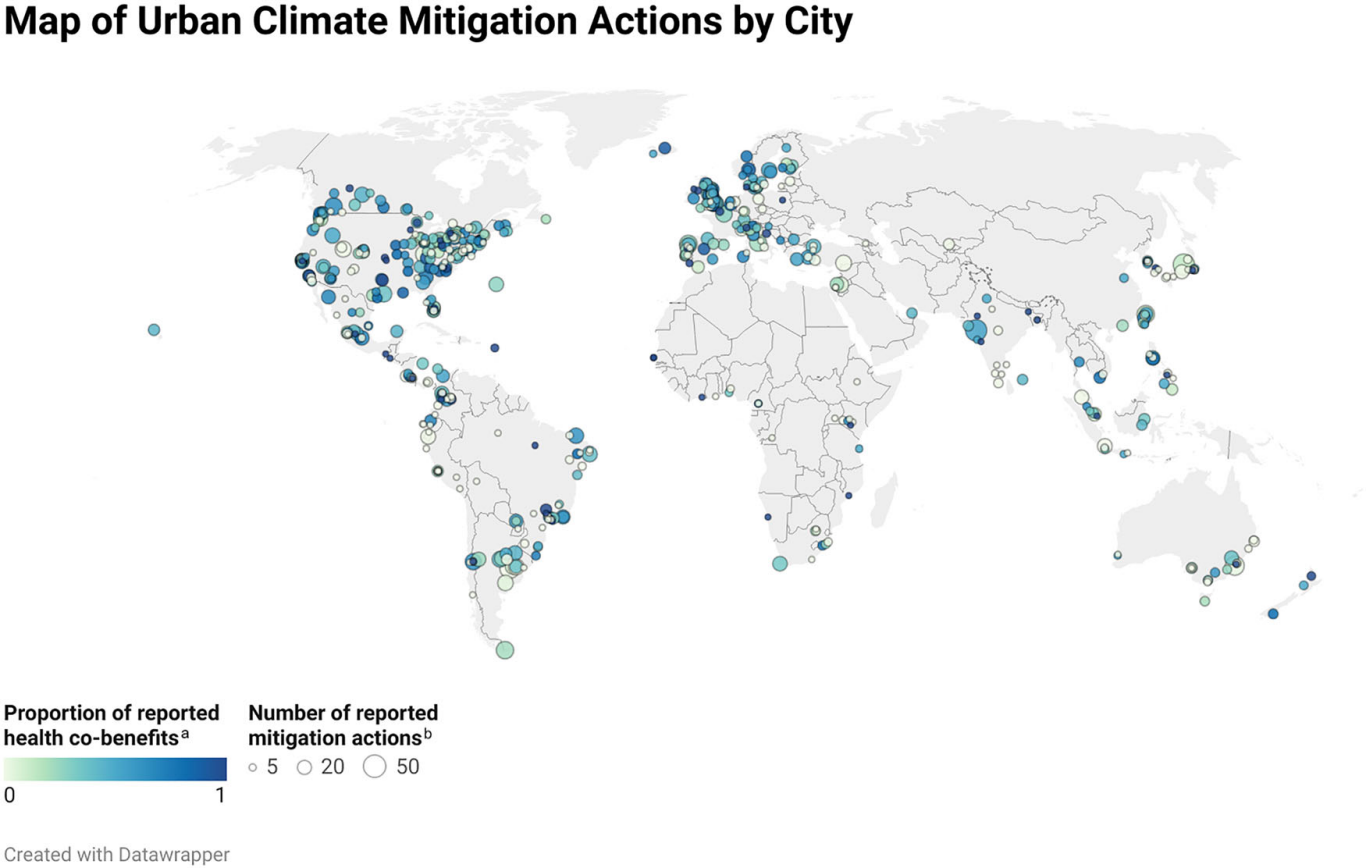
World Map of climate mitigation actions by city. **a** Colour palette indicates the 'Proportion of reported health co-benefits’ compared to the number of total actions reported from one city (0, light green indicates no health co-benefits were reported for any action and 1, dark blue indicates that for all actions health co-benefits were reported). **b** The size of the dots indicates the 'Number of reported mitigation actions' the city reported to CDP.

Most actions (in total numbers) with no or very low proportion of reported health co-benefits were reported by cities from Latin America, North America and Europe (Fig. 1). We also found that most cities that report no health co-benefits only report one mitigation action, with some outlier cities that report no or a low proportion of health co-benefits but a large number of mitigation actions.

We used the country grouping developed by the DAC (Development Assistance Committee) at OECD from 2022^19^ to categorise the countries by income level. Most actions were reported from high-income countries (HIC) (2776, 72.4%), with 27.6% of the actions (1059) being reported from low-and middle-income countries (LMIC) (176 cities from middle-income countries and 10 cities from least developed and low-income countries). The energy (1661, 43.3%), and transportation sectors (1154, 30.1%), were most frequently associated with reported urban mitigation action while the least actions were reported from the AFOLU (Agriculture, Forestry and Other Land Use and Food) (326, 8.5%) and industry sectors (58, 1.5%). Most actions had not been implemented (1285, 33.5%) and were in pre-operation (2550, 66.5%).

Co-benefits were reported for a high proportion of the mitigation actions analysed (3366, 87.8%). The most frequently reported co-benefits were economic (2328, 34.6%), followed by social (2037, 30.2%), and health co-benefits (1488, 22.1%). Within health co-benefits, ‘Improved air quality’ (972, 36.8%) and ‘Improved mental wellbeing/ quality of life’ (346, 13.1%) were the most reported whereas ‘Increased food security’ (72, 2.7%) and ‘Reduced disaster/ disease/contamination-related health impacts’ (67, 2.5%) were the least reported health co-benefits together with ‘Improved public health’ benefits (21, 0.8%).

### Factors relating to the reporting of co-benefits of mitigation actions

**Fig. 2:**
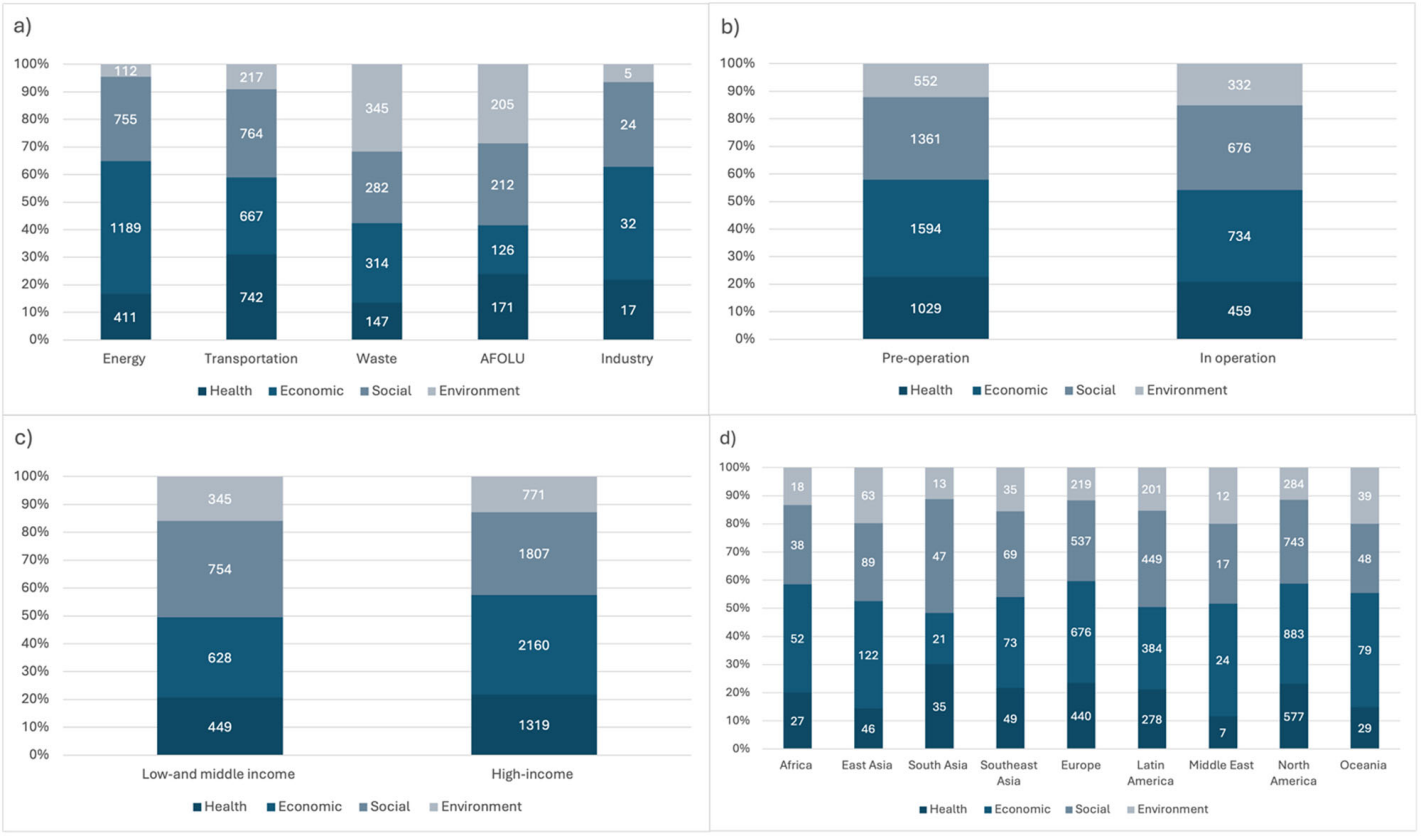
Distribution of economic, social, health, and environment co-benefits across sectors, regions, status, and country-income. **a** depicts reported co-benefits by sector, **b** depicts reported co-benefits by implementation status, **c** depicts reported cobenefitsby country-income and (**d**) depicts co-benefits by region.

Reported co-benefits varied across sectors, regions, country income and implementation status, with economic and social co-benefits being the most commonly reported co-benefits. In the transport sector, social (764, 66.2%) and health (742, 64.3%) co-benefits were most commonly reported compared to all other sectors where health co-benefits were the least or second least reported co-benefits (Fig. 2). In the waste sector, environmental (345, 54.2%) co-benefits were most reported, and in the AFOLU sector social (212, 65%) co-benefits were most reported. Most actions in the energy and industry sector reported economic and social co-benefits (economic 1189, 71.6%; social 755, 45.5% in the energy sector, and economic 32, 55.2%; social 24, 41.4% in the industry sector) respectively.

Across all regions economic and social co-benefits were the top two types of co-benefits reported, except South Asia, where social (47, 59.5%) and health co-benefits (35, 44.3%) were most reported.

Little difference was found between co-benefit types according to whether actions were in operation or pre-operation. Cities from LMIC were most likely to report social (LMIC 754, 71.2%, HIC 1807, 65.1%) co-benefits, whereas cities from HIC were most likely to report economic (HIC 2160, 77.8%, LMIC 628, 59.3%) co-benefits.

Furthermore, actions that are in ‘pre-operation’ are more likely to report health co-benefits (*OR* = 1.22, *p* = *0.038*) compared to actions ‘in operation’ and no significant differences were found among regions. Actions from high-income countries were less likely to report health co-benefits (*OR* = 0.49, *p* < *0.001*) and significant differences were found across actions from different sectors. Actions from energy (*OR* = 0.2 *p* < *0.001*), industry (*OR* = 0.3, *p* = *0.001*), and waste (*OR* = 0.21, *p* < *0.001*) were significantly less likely, whereas actions from transport sector (*OR* = 1.63, *p* = *0.002*) were significantly more likely to report health co-benefits compared to actions from the AFOLU sector. See Supplementary information 7.

### Reporting of health co-benefits across sectors and status

**Fig. 3:**
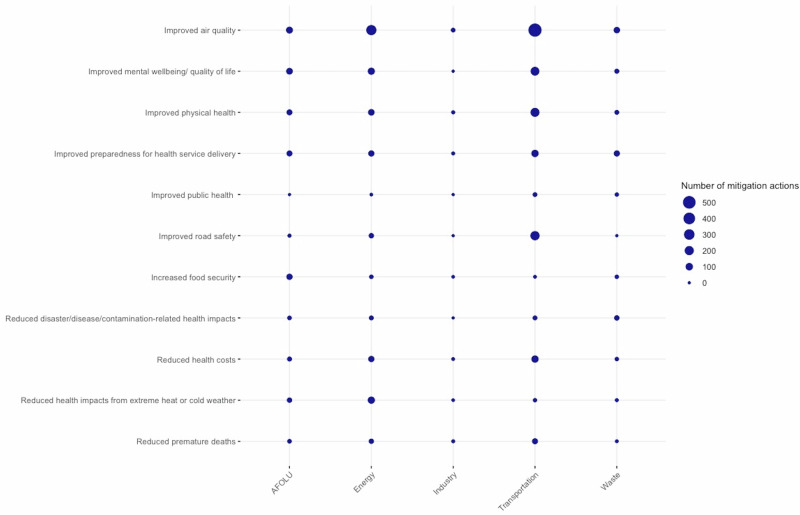
Reported health co-benefits per sector. **a** The figure shows the distribution of all individual health co-benefits by sector. **b** The size of the bubble depicts the number of mitigation actions from the respective sector that reported the respective health co-benefit.

The types of health co-benefits reported also differed across sectors, although improved air quality was commonly noted as a co-benefit across multiple sectors. ‘Improved air quality’ (76, 23.3%) and ‘Improved mental health/ wellbeing’ (68, 20.9%) were the most commonly reported health co-benefits for the AFOLU sector. The most frequently reported health co-benefits in the transport sector were ‘Improved air quality’ (556, 48.2%) and ‘Improved road safety’ (202, 17.5%). For the energy sector ‘Improved air quality’ (270, 16.3%), and ‘Reduced health impacts from extreme heat or cold weather’ (89, 5.4%) were reported the most, while in the waste sector ‘Improved air quality’ (58, 9.1%), and ‘Improved preparedness for health service delivery’ (49, 7.7%) were the most common reported health co-benefits. Actions from Industry reported health co-benefits the least, but ‘Improved air quality’ (12, 20.7%) and ‘Improved physical health’ (4, 6.9%) were most commonly reported (Fig. 3).

**Fig. 4:**
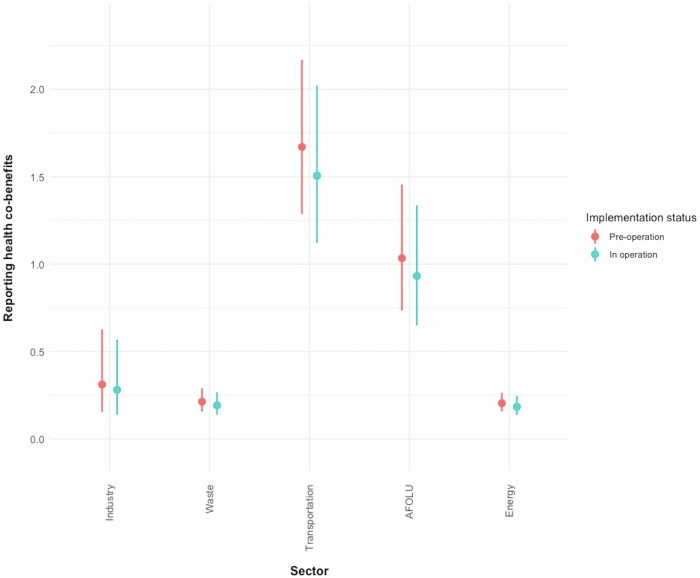
Odds ratio of reporting of health co-benefits by sector and implementation status. **a** Predicted probabilities derived from the generalized linear mixed effects model (GLME) ± 95% confidence intervals. **b** Blue colour indicates pre-operation, red colour indicates in operation.

The predicted odds of reporting health co-benefits were highest for actions from the transport sector (GLMM, pre-operation: OR = 1.67, CI 1.29–2.17; in operation: OR = 1.51, CI 1.12–2.02) and from AFOLU (GLMM pre-operation: OR = 1.03, CI 0.73 – 1.46; in operation: OR = 0.93, CI 0.65–1.34). The odds for reporting health co-benefits were lowest for actions from the waste sector (GLMM, pre-operation: OR = 0.21, CI 0.16–0.3; in operation: OR = 0.2, CI 0.14–0.27) (Fig. 4). There was no significant association between reporting health co-benefits and the population size of a city. Whilst the odds of reporting health co-benefits were consistently higher for actions pre-operation, there was no significant association. Supplementary information 8 includes details about the model specification and results.

### City characteristics linked to reporting a high proportion of health co-benefits

A few cities reported a large number of actions with minimal or no reporting of health co-benefits. We therefore tested whether region, country-income or population size was associated with cities reporting a high proportion of health co-benefits compared to the total number of actions reported.

Among the 584 cities, the highest number of reported mitigation actions per city was *n* = 46, the lowest *n* = 1, and the median of reported actions was *n* = 4. The highest proportion of reported health co-benefits was 1, and the lowest 0. A binomial model showed that population size (*z* = 3.55, *p* < 0.001) was significantly associated with cities reporting a high proportion of health co-benefits. Cities from Africa (*z* = −2.02, *p* < 0.5) and the Middle East (*z* = −2.16, *p* < 0.5) were significantly less likely to report a high proportion of health co-benefits. Country-income was not significantly associated with reporting a high proportion of health co-benefits (Supplementary Information 9).

## Discussion

The CDP-ICLEI Track platform provides an unparalleled opportunity to monitor urban climate policies and their implementation in diverse locations, with the potential to evaluate the role of co-benefits in accelerating climate action. As a major source of national GHG emissions, cities play an important role in delivering agreed national mitigation targets under the Paris Agreement.

Overall, we find that while most cities reported co-benefits related to their climate mitigation activities, many failed to report or only conducted minimal reporting of health co-benefits. It is positive that many cities are demonstrating good awareness and reporting of ancillary benefits associated with climate action, but these often focus on economic and social benefits. For almost 70% of all reported actions with co-benefits economic co-benefits were reported, whereas health co-benefits were reported for only 44%, suggesting a gap between the potential co-benefits and recognised health co-benefits of mitigation actions.

Across all sectors, economic and social co-benefits were most reported. Health co-benefits were reported second to social benefits in the transportation sector, suggesting that health benefits of transport interventions, such as increased physical activity and improved air quality, are better known than the health benefits of interventions in other sectors. Actions in the waste sector showed few reported health co-benefits despite the opportunity for sustainable waste management interventions to result in substantial health benefits through reduced air pollution^20,21^. This highlights a gap in awareness and the need to raise understanding of the health pathways of mitigation actions in many sectors.

We did not find large differences across regions in reporting rates for health co-benefits. We found that across all regions, except for South Asia, economic co-benefits were the most reported co-benefits and that LMIC reported social co-benefits most, whereas HIC reported economic co-benefits most. In contrast to existing evidence that LMIC demonstrated greater health engagement in their nationally determined contributions (NDCs)^22^, HIC were more likely to report health co-benefits compared to LMIC. Future research could investigate the reasons why LMIC demonstrate greater health engagement in their NDCs, but at a city-wide level are less likely to report the health co-benefits of mitigation actions compared to cities from HIC. Whilst this analysis focussed solely on mitigation actions, both types of actions, i.e., adaptation and mitigation should be integrated to achieve synergies and minimise risks of maladaptation, especially in LMIC which experience a disproportionate burden of the impacts of climate change.

When comparing individual health co-benefits, we found that ‘Improved air quality’ (which is an exposure rather than a health outcome, for more information see Supplementary Information 1) was by far the most reported health co-benefit across all sectors and in all regions was among the top two reported health co-benefits. This is in line with existing research on climate and health literature showing that health topics are dominated by air quality studies^16^.

We also found that selected cities reported many actions across sectors with economic, social, or environmental co-benefits, but did not report any or a very low proportion of health co-benefits. Population size had a small but significant association with reporting a large proportion of health co-benefits. Cities from Africa and the Middle East were significantly less likely to report a high proportion of health co-benefits. Future research should aim to understand why these cities do not recognise specifically the health co-benefits of their mitigation actions, whether this is a reporting error or whether more awareness about the pathways and how health benefits are linked to mitigation actions is required.

The dominance of reported actions from North America (*n* = 1322) and Europe (*n* = 1050) and the scarce data, particularly from Africa (*n* = 73), South Asia (*n* = 79) and the Middle East (*n* = 43) is particularly notable. There was limited inclusion from China and India. Only 21 out of the total 5040 actions were reported from Chinese cities, which were excluded from the analysis due to missing information. 91 out of the 5040 actions were reported from Indian cities out of which 58 actions were excluded due to missing information. Given these two countries are the most populous in the world, accounting for around 35% of the world’s population, future research should include more cities from the two countries.

We also found large amounts of missing data for the implementation status of the action and sector, and had to omit 1205 actions and 208 cities from the analysis. Most omitted actions (68%) were from HIC, introducing potential selection bias. Further investigation is needed to find out why a high proportion of cities from Latin America (28%) did not report the status or sector of their mitigation actions and why missing data were frequent in the energy sector (24%). The uneven regional distribution and missing data of certain sectors and implementation stages restrict the generalisability of our findings.

The CDP data are highly variable, probably because the individuals assigned to complete the CDP questionnaire vary widely, ranging from interns to senior members of the climate team. This variability in knowledge, along with the potential for human error, can affect the accuracy of the data and the findings of this study, which necessitates a cautious interpretation of the study’s conclusions. Furthermore, the CDP-ICLEI Track platform includes a feature that allows data to be copied forward, saving time. As the questionnaire evolves each year, small changes or additional columns and drop-down options can sometimes be overlooked, resulting in an incomplete response. We further note that the CDP response options for health co-benefits of mitigation actions, such as ‘Reduced health impacts from extreme heat or cold weather’ could be adaptation and/or mitigation benefits depending on the primary objective. Underlining environmental factors and urban design can further impact the perception and delivery of health co-benefits in cities. For example, switching to renewable energy and the associated improvements of air quality are more pronounced and more perceived in cities where current air pollution is high, which in turn may impact the awareness of health co-benefits. Improvements in awareness and reporting of data would greatly enhance the potential contribution of urban actions to the achievement of the climate goals of the Paris Agreement by capitalising on the health and other co-benefits of climate change mitigation policies.

This paper demonstrated that there is a lack of reporting on health co-benefits of climate actions from the energy and waste sectors, regardless of which region of the world the action was undertaken. Although a small trend was evident, there was no significant difference in reporting health benefits between actions in the pre-implementation stage compared to those where actions were underway. Our findings underscore the importance of enhancing awareness among urban policymakers regarding the potential health co-benefits that can be achieved, particularly in sectors where these health connections may be less apparent. Moreover, this paper underscores the need to improve reporting standards on health co-benefits. An improved understanding and monitoring of these health co-benefits by local governments can facilitate the implementation of ambitious climate action. Currently, the scarcity of city-level data from LMIC presents a significant challenge for effectively monitoring urban climate initiatives in regions where most of the future urban growth will take place. Further research is needed to understand the disparities among cities in reporting the health co-benefits of climate mitigation efforts.

## Methods

CDP Cities is an initiative by CDP (formerly the Carbon Disclosure Project) that provides a platform for cities around the world to report their environmental impact data on an annual basis. The data include information on greenhouse gas emissions, climate hazards and vulnerability, adaptation and mitigation plans and actions, as well as more specific topics such as water, energy, buildings, transport, and waste. With its platform, CDP aims to promote transparency by encouraging cities to disclose their data. It helps cities to track their progress on climate action and can identify areas in need of improvement. By collecting and analysing data from numerous cities, CDP provides benchmarks that cities can use to measure their performance against other cities.

Since 2018, CDP has been scoring cities based on the completeness of their responses and their performance on climate action. Cities receive a score (A, B, C, or D) through a scorecard at the end of the disclosure cycle, along with personalised feedback. The scores are private, except cities that receive an A are made public and featured on the annual Cities A List. In 2022, 122 cities scored an A out of which 90 cities (74%) were from Canada, the United States, and Europe. Country, country-income, and region could act as potential confounders, and we therefore did not include city scoring as a potential correlate of reporting health co-benefits. The data reported to CDP, which are publicly available, are completed by city representatives. To identify potential correlates of cities reporting on health co-benefits, we used data reported by cities to CDP in 2022. Local governments reporting to CDP comprise a variety of local entities, including municipalities, cities, local counties, and boroughs. We used data from all local governments and referred to all as ‘cities’.

### Response data

Cities were asked to report on their adaptation and mitigation actions, but for the purpose of this study, we solely focused on the mitigation actions data (Question 9.1 in the 2022 questionnaire). Cities were able to report multiple climate mitigation actions and select multiple co-benefits for one action from a drop-down menu. Cities were asked to “*Describe the outcomes of the most significant mitigation actions your jurisdiction is currently undertaking*”.

One response option was ‘Co-benefits realised’, where cities can choose from a drop-down menu of a total of 37 co-benefits (11 economic, 10 social, 8 public health, 6 environmental, 2 other impacts measured). The full co-benefits list can be found in Supplementary Information 2. All categories of co-benefits were included (economic, social, health, and environmental), but as we were interested in the individual health co-benefits, we selected the response ‘Public Health’ for our dependent variable. Several steps were conducted to prepare the data for analysis. The response options for the question on co-benefits changed from the previous year, which is why many co-benefits were labelled as ‘Other’ even though they fitted in one of the existing co-benefits categories: economic, social, health, and environment. Key word searches based on the response options from 2021 were conducted to match the 360 ‘Other’ responses with one of the four co-benefits. Through this exercise an additional 158 co-benefits were included: Economic (*n* = 20), health (*n* = 21), social (*n* = 110) and environmental (*n* = 7). ’Improved public health’ (*n* = 21) is a response option from the 2021 CDP Cities questionnaire, which explains the small number of data points. A full list of the co-benefit response options from the 2021 questionnaire and the keyword searches can be found in Supplementary Information 3.

For our outcome variable, we created a new binary variable called ‘health co.benefits’ with N = 0 and Y = 1.

### Predictor variables

*Country income*: Data on country income was obtained from the DAC (Development Assistance Committee) OECD country grouping from 2022^19^, which is based on gross national income (GNI) per capita, published by the World Bank. We created a new variable called ‘Country income’. Due to small sample sizes, we merged middle-income and low-income countries to low-and middle-income countries (*n* = 1443).

*Sector of climate mitigation action*: For the sectoral action data, we identified large data gaps. 830 out of 5040 actions had no reported sector. We analysed these 830 actions and based on the action description and action type we matched an additional 728 actions to a response option. During this process, we identified 8 cross-sectoral actions, which we omitted from the analysis, despite their great importance for transformative change. We also omitted any ‘Other’, ‘NA’ or ‘No mitigation action in place’ data entries. We further omitted 804 (16%) actions from the analysis due to missing status data. In total we omitted 1215 (24%) mitigation actions, where either the status, the sector, or both were missing. A list of all primary emission sectors can be found in Supplementary Information 4.

*City population size*: Cities reported information on population size. This information was missing for 77 cities and was manually entered using publicly available data from websites census.gov, citypopulation.de, and wikipedia.org. A list of the 77 cities and sources is provided in Supplementary Information 5.

*Implementation status*: To facilitate analysis, we merged the 13 different implementation status categories provided by the CDP-ICLEI track questionnaire into two: ‘pre-operation’ and ‘in operation’. A full list of all implementation categories can be found in Supplementary Information 4.

*Region*: We largely adapted the regional categorisation from CDP, with the exception of merging ‘United States of America’ and ‘Canada’ into the region ‘North America’. All regional categories can be found in Supplementary Information 4.

For the statistical analysis, we recoded all responses of our outcome variables to numerical values between 0 and 5, because CDP data, apart from population size, consist of non-numerical values. Due to large amounts of missing information, data on ‘Reported emission reductions’, ‘Start/ End year of action’, ‘Total costs of the action’, and ‘Finance status’ were not included in the analysis. The remaining data fields from the mitigation action dataset were not included in the analysis as they were not of relevance for this analysis. The full mitigation questionnaire and all data fields for Question 9.1 can be found in Supplementary Information 6.

### Statistical analysis

All statistical analyses were performed using R Statistical Software (v. 4.3.0)^23^. We initially conducted univariate analyses to test the variables status, country-income, sector, region, and population (Supplementary Information 7). Because one city can report multiple mitigation actions, actions are not independent but clustered and can be similar in terms of population size, country GDP, as well as national policies. To account for the clustering, we used a generalized linear mixed-effects model (GLMM) in R^23^. We used the *glmer* function with the binomial family and logit link function in lme4 package^24^. The explanatory variable ‘population’ was scaled before the analysis to facilitate the interpretation of the model output. Our GLMM used health co-benefits reported as response variable, and controlled for status, sector, and population with country and city ID number as random effects.

To assess city characterises linked to reporting a high proportion of health co-benefits, a binomial analysis was conducted. We created a sub-dataset where we merged all reported actions of one city into one data point and created new variables stating the total number of actions reported by each city and the proportion of reported health co-benefits compared to the number of reported mitigation actions per city. The analysis had a sample size of 584 cities. To assess whether region, country-income, and population were associated with reporting a large proportion of health co-benefits, we used a binomial generalized linear model with large proportion of health co-benefits as response variable and controlled for population, region, and country-income.

## ^Supplementary information^

Supplementary Information_Anton et al._Revised_241025.

## Data Availability

The data analysed during this study is publicly available on the CDP website: [https://data.cdp.net].

## Code availability

The underlying code for this study is not yet publicly available but will be made available to qualified researchers and on reasonable request from the corresponding author.
