# Supplementary material for: Pathways to health: Reporting on health co-benefits from urban climate mitigation action varies by sector
Source: NPJ Urban Sustain. 2025 Nov 29;6(1):9. doi: 10.1038/s42949-025-00311-y (PMC12795749; doi:10.1038/s42949-025-00311-y)
Supplement: Supplementary file 1 — Supplementary Information_Anton et al._Revised_241025. [file 42949_2025_311_MOESM1_ESM.pdf]

Pathways to health: Reporting on health co-benefits from urban climate mitigation action varies by sector and status

Supplementary Information

1. Background information 2

2. Full list of all co-benefit categories from the 2022 CDP- ICLEI Track Cities Mitigation Questionnaire 3

3. Full list of co-benefit categories from the 2021 questionnaire and list of key word searches 4

4. Merging and recoding variables 5

5. List of cities with missing population data and sources 6

6. Full CDP-ICLEI Track Mitigation action questionnaire 2022 8

7. Univariate analysis 9

8. Analyses of main findings 12

9. City characteristics linked to reporting a high proportion of health co-benefits 14

1. Background information

Climate mitigation interventions, such as replacing fossil fuels with renewables, promoting public transport and active travel, and nature-based solutions (e.g. urban trees and green infrastructure) can lead to health co-benefits such as lower rates of cancer, reduced respiratory disease, lower rates of obesity, better mental health and reduced cardiovascular disease. The three main pathways by which mitigation actions can yield health co-benefits are: reduction of air pollution, transition to a healthy and sustainable diet, and increased physical activity from active travel.

When assessing the health co-benefits of mitigation actions, we further differentiate between health exposures and health outcomes. Under health exposure we define any measurable characteristic that can affect human health. Exposures include both environmental factors including exposure to air pollution or access to green space as well as behaviours including physical activity or diets. We define health outcomes as any measurable disease, disability, or health state. This includes for example, death or disease burden. The CDP-ICLEI Track questionnaire co-benefit response options include both health exposures (e.g. improved air quality) and health outcomes (e.g. reduced premature deaths). While it is desirable to measure and report health outcomes, they are often slow to respond. Changes in health-related exposures can be measured and reported alongside known exposure-response curves to model health outcomes.

2. Full list of all co-benefit categories from the 2022 CDP- ICLEI Track Cities Mitigation Questionnaire

Economic

- Job creation
- Revenue generation
- Reduced costs
- Increased energy security
- Business/ technological innovation
- Increased labour productivity
- Improved labour conditions
- Increased economic production
- Reduced natural resource depletion
- Reduced congestion
- Reduced disruption of energy, transport, water and communications networks

Social

- Increased water security
- Increased food security
- Improved mobility and access
- Improved road safety*
- Reduced fuel poverty*
- Increased security/ protection for poor/ vulnerable populations
- Increased social inclusion, equality and justice
- Increased transparency and accountability
- Improved education and public awareness
- Enhanced climate change adaptation
- Enhance resilience to shocks and disasters

Public Health

- Improved physical health
- Improved mental wellbeing/ quality of life
- Improved air quality
- Improved preparedness for health service delivery
- Reduced health impacts from extreme heat or cold weather
- Reduced disaster/ disease/ contamination-related health impacts
- Reduced premature deaths
- Reduced health costs

Environmental

- Improved water/ soil quality
- Improved waste management Reduced noise/ light pollution
- Reduced noise/ light pollution
- Increased/ improved green space
- Protected/ improved biodiversity and ecosystem services

Other impacts measured

- Other impacts from climate actions
- Do not know

*We re-coded these social co-benefits as health co-benefits.

3. **Full list of co-benefit categories from the 2021 questionnaire and list of key word searches**

Co-benefit categories from the 2021 CDP-ICLEI Questionnaire

- Disaster Risk Reduction
- Enhanced resilience
- Disaster preparedness
- Enhanced climate change adaptation
- Reduced GHG emissions
- Improved resource efficiency (e.g. food, water, energy)
- Poverty reduction/eradication
- Social inclusion, social justice
- Social community and labour improvements
- Greening the economy
- Economic growth
- Promote circular economy
- Job creation
- Improved resource quality (e.g. air, water)
- Improved public health
- Improved resource security (e.g. food, water, energy)
- Security of tenure
- Resource conservation (e.g. soil, water)
- Ecosystem preservation and biodiversity improvement
- Improved access to and quality of mobility services and infrastructure
- Shift to more sustainable behaviours
- Improved access to data for informed decision-making

Key word search to match the co-benefit categories from the 2021 questionnaire to the co-benefit categories used in our analysis:

- **Economic co-benefits:**
- "Economic", "economy", "greening", "Greening", "growth", "circular", "creation", "Job", "job", "Employment", "employment", "growth", "economic", "economy"

- **Social co-benefits:**
- "Social", "risk", "resilience", "preparedness", "adaptation", "efficiency", "poverty", "Poverty", "justice", "community", "resource", "tenure", "mobility", "behaviours", "data"

- **Environment co-benefits:**
- "Environment", "conservation", "preservation", "Conservation", "Preservation", "environment"

- **Health co-benefits:**
- "Health", "air", "health", "Mental", "wellbeing", "Air", "Physical", "physical", "Extreme weather", "weather", "Health service", "health service", "Disaster", "disease", "disaster", "premature", "Premature", "road safety", "Road safety", "health costs", "Health costs", "road safety", "Road safety", "food security", "Food security", "public health"

4. Merging and recoding variables

Region:

<table>
<tr><th>CDP-ICLEI Track Questionnaire 2022</th><th>Categorisation for analysis</th></tr>
<tr><td>Africa</td><td>Africa</td></tr>
<tr><td>Middle East</td><td>Middle East</td></tr>
<tr><td>South Asia</td><td>South Asia</td></tr>
<tr><td>East Asia</td><td>East Asia</td></tr>
<tr><td>Southeast Asia</td><td>Southeast Asia</td></tr>
<tr><td>Oceania</td><td>Oceania</td></tr>
<tr><td>Europe</td><td>Europe</td></tr>
<tr><td>Latin America</td><td>Latin America</td></tr>
<tr><td>United States of America</td><td rowspan="2">North America</td></tr>
<tr><td>Canada</td></tr>
</table>

Status of action in the reporting year:

<table>
<tr><th>CDP-ICLEI Track Questionnaire 2022</th><th>Categorisation for analysis</th></tr>
<tr><td>Pre-implementation
• Scoping
• Pre-feasibility study
• Feasibility finalized, but currently no finance secured
• Feasibility finalized, and finance partially secured
• Feasibility finalized, and finance fully secured</td><td rowspan="2">Pre-operation</td></tr>
<tr><td>Implementation
• Implementation complete in the reporting year
• Implementation underway with completion expected in less than one year
• Implementation underway with completion expected in more than one year</td></tr>
<tr><td>Post-implementation/ Operation
• Action in operation (jurisdiction-wide)
• Action in operation (across most of jurisdiction)
• Action in operation (targeted to sector/ location)
• Other, please specify</td><td>In operation</td></tr>
</table>

Primary emissions sector:

<table>
<tr><th>CDP-ICLEI Track Questionnaire 2022</th><th>Categorisation for analysis</th></tr>
<tr><td>Transportation</td><td>Transportation</td></tr>
<tr><td>Waste</td><td>Waste</td></tr>
<tr><td>Industrial Processes and Product</td><td>Industry</td></tr>
<tr><td>Stationary energy</td><td rowspan="2">Energy</td></tr>
<tr><td>Generation of grid-supplied energy</td></tr>
<tr><td>Agriculture, Forestry and Land Use</td><td rowspan="2">AFOLU</td></tr>
<tr><td>Food</td></tr>
<tr><td>Other sectoral action</td><td rowspan="3">Omitted</td></tr>
<tr><td>No mitigation action in place</td></tr>
<tr><td>Other, please specify</td></tr>
</table>

5. List of cities with missing population data and sources

City of Beverly, MA	https://www.census.gov/quickfacts/fact/table/beverlycitymassachusetts/NES010220
Kami-Amakusa City	https://en.wikipedia.org/wiki/Kami-Amakusa
Yokkaichi City	https://www.citypopulation.de/en/japan/mie/
Umhlathuze	https://municipalities.co.za/demographic/1110/city-of-umhlathuze-local-municipality
Takashima City	https://citypopulation.de/en/japan/admin/shiga/25212__takashima/
Kani City	https://www.citypopulation.de/en/japan/gifu/_/21214__kani/
Kan-onji City	https://www.citypopulation.de/en/japan/kagawa/_/37205__kanonji/
Shinjuku City	https://citypopulation.de/en/japan/tokyocity/13104__shinjuku_ku/
Lund Municipality	https://www.citypopulation.de/en/sweden/admin/skåne/1281__lund/
Municipalidad Distrital de Jesús María	https://es.wikipedia.org/wiki/Distrito_de_Jesús_María
Nagakute City	https://citypopulation.de/en/japan/admin/aichi/23238__nagakute/
Prefeitura de Quissamã	https://cidades.ibge.gov.br/brasil/rj/quissama/panorama
Bani-Suhaila Municipality	https://www.pcbs.gov.ps/Downloads/book2364-1.pdf
Lubumbashi	https://populationstat.com/democratic-republic-of-the-congo/lubumbashi
Kagoshima	https://www.citypopulation.de/en/japan/kagoshima/_/46201__kagoshima/
Salt Lake City, UT	https://data.census.gov/profile?g=160XX00US4967000
Aira City	https://www.citypopulation.de/en/japan/kagoshima/_/46225__aira/
Matsue City	https://www.citypopulation.de/en/japan/shimane/_/32201__matsue/
Town of Durham, NH	https://www.census.gov/quickfacts/fact/table/durhamtownstraffordcountynewhampshire/PST045222
Shibata City	https://www.citypopulation.de/en/japan/niigata/_/15206__shibata/
Fukuyama City	https://www.citypopulation.de/en/japan/hiroshima/_/34207__fukuyama/
Oda City	https://www.citypopulation.de/en/japan/shimane/_/32205__ōda/
Ureshino City	https://www.citypopulation.de/en/japan/saga/_/41209__ureshino/
Town of Guilford, VT	https://data.census.gov/profile/Guilford_town,_Windham_County,_Vermont?g=060XX00US5002530925
Myoko City	https://www.citypopulation.de/en/japan/admin/niigata/15217__myōkō/
Kurashiki City	https://www.citypopulation.de/en/japan/okayama/_/33202__kurashiki/
Yamanashi City	https://www.citypopulation.de/en/japan/yamanashi/_/19205__yamanashi/
City of Shizuoka	https://www.citypopulation.de/en/japan/shizuoka/_/22100__shizuoka/
Konan City	https://www.citypopulation.de/en/japan/shiga/_/25211__konan/
Inazawa City	https://www.city.inazawa.aichi.jp/0000000616.html
Kikugawa City	https://www.citypopulation.de/en/japan/shizuoka/_/22224__kikugawa/
Hanamaki City	https://www.citypopulation.de/en/japan/iwate/_/03205__hanamaki/
Ogori City	https://www.citypopulation.de/en/japan/admin/fukuoka/40216__ogōri/
Embu City	https://en.wikipedia.org/wiki/Embu,_Kenya
Hokuto City	https://www.citypopulation.de/en/japan/yamanashi/_/19209__hokuto/
Tainai City	https://www.citypopulation.de/en/japan/admin/niigata/15227__tainai/
Town of Wellfleet, MA	https://data.census.gov/profile/Wellfleet_town,_Barnstable_County,_Massachusetts?g=060XX00US2500174385
Tagajo City	https://www.citypopulation.de/en/japan/admin/miyagi/04209__tagajō/
Shima City	https://www.citypopulation.de/en/japan/mie/_/24215__shima/
Iwakura City	https://citypopulation.de/en/japan/admin/aichi/23228__iwakura/
Kato City	https://www.citypopulation.de/en/japan/admin/hyōgo/28228__katō/
Hachioji	https://www.citypopulation.de/en/japan/tokyo/_/13201__hachiōji/
Setouchi City	https://www.citypopulation.de/en/japan/okayama/_/33212__setouchi/
Asakura City	https://citypopulation.de/en/japan/admin/fukuoka/40228__asakura/
City of Miyakojima	https://www.citypopulation.de/en/japan/okinawa/_/47214__miyakojima/
Nihonmatsu City	https://www.citypopulation.de/en/japan/fukushima/_/07210__nihonmatsu/
City of Iwanuma	https://www.citypopulation.de/en/japan/miyagi/_/04211__iwanuma/
Minamikyushu City	https://www.citypopulation.de/en/japan/kagoshima/_/46223__minamikyūshū/

Tamana City	https://www.citypopulation.de/en/japan/kumamoto/_/43206__tamana/
Nanjo City	https://www.citypopulation.de/en/japan/okinawa/_/47215__nanjō/
Hirosaki City	https://www.citypopulation.de/en/japan/aomori/_/02202__hirosaki/
Saiki City	https://www.citypopulation.de/en/japan/oita/_/44205__saiki/
Takayama City	https://www.citypopulation.de/en/japan/gifu/_/21203__takayama/
Koshigaya City	https://www.citypopulation.de/en/japan/saitama/_/11222__koshigaya/
City of Sakuragawa	https://www.citypopulation.de/en/japan/admin/ibaraki/08231__sakuragawa/
The City of Suzuka	https://www.citypopulation.de/en/japan/mie/_/24207__suzuka/
Ibaraki City	https://www.citypopulation.de/en/japan/osaka/_/27211__ibaraki/
Hashimoto City	https://www.citypopulation.de/en/japan/wakayama/_/30203__hashimoto/
Adachi City	https://www.citypopulation.de/en/japan/tokyocity/13121__adachi_ku/
City of Tsukuba	https://www.citypopulation.de/en/japan/ibaraki/_/08220__tsukuba/
Ube City	https://www.citypopulation.de/en/japan/yamaguchi/_/35202__ube/
Sanyo-Onoda City	https://www.citypopulation.de/en/japan/yamaguchi/_/35216__sanyō_onoda/
Chikuma City	https://www.citypopulation.de/en/japan/nagano/_/20218__chikuma/
San Rafael de Heredia	https://citypopulation.de/en/costarica/urbandistricts/heredia/40501__san_rafael/
Murakami City	https://www.citypopulation.de/en/japan/niigata/_/15212__murakami/
Municipalidad de Valparaíso	https://citypopulation.de/en/chile/mun/admin/valparaíso/05101__valparaíso/
Nagasaki City	https://www.citypopulation.de/en/japan/nagasaki/_/42201__nagasaki/
Takarazuka City	https://www.citypopulation.de/en/japan/hyogo/_/28214__takarazuka/
Izumo City	https://www.citypopulation.de/en/japan/shimane/_/32203__izumo/
Steve Tshwete	https://www.citypopulation.de/en/southafrica/admin/mpumalanga/MP313__steve_tshwete/
Mine City	https://www.citypopulation.de/en/japan/admin/yamaguchi/35213__mine/
Fukuchiyama City	https://www.citypopulation.de/en/japan/kyoto/_/26201__fukuchiyama/
Kitahiroshima City	https://www.citypopulation.de/en/japan/hokkaido/_/01234__kitahiroshima/
Shiki City	https://www.citypopulation.de/en/japan/admin/saitama/11228__shiki/
San Antonio	https://en.wikipedia.org/wiki/San_Antonio_Province
Saijo City	https://www.citypopulation.de/en/japan/ehime/_/38206__saijō/

6. Full CDP-ICLEI Track Mitigation action questionnaire 2022

Screenshots of the 2022 CDP-ICLEI Track Mitigation actions questionnaire. Full 2022 Cities Questionnaire is available online here: https://guidance.cdp.net/en/guidance?cid=37&ctype=theme&idtype=ThemeID&incchild=1&microsite=0&otype=Questionnaire&tags=TAG-637%2CTAG-13013

Mitigation Actions

(9.1) Describe the outcomes of the most significant mitigation actions your jurisdiction is currently undertaking. Note that this can include those in the planning and/or implementation phases.

A selection must be made for both fields in Column 1. Please note your data won't be saved if one field is left blank.

Questionnaire Pathway

- **Pathway 1:** Column 1 - 10
- **Pathway 2 and 3:** Column 1 - 13

Connection to other frameworks

- **GCoM:** Mitigation Pillars^
- **TCFD:** Metric and Targets (Disclosure A)
- **Race to Zero**
- **European Climate Pact**
- **Sustainable Development Goals:** SDG11, SDG13

Response Options

Please complete the following table. You are able to add rows by using the "Add Row" button at the bottom of the table.

Primary emissions sector addressed and action type^	Action description and web link to further information^	Start year of action	Year for which mitigation is expected to last	Impact indicators measured^
Select from:<br><br>Appendix C (Mitigation actions)	Text field	Numeric field	Select from:<br>• Drop-down list of years (2022-2050, 2051 or later)<br>• End year not known/not applicable	Select all that apply:<br>• Estimated annual emissions reductions due to action<br>• Estimated annual energy savings due to action<br>• Estimated annual renewable energy generated due to action<br>• None of the above impacts associated with this action have been measured<br>• Other impact indicator, please specify

Estimated annual emissions reductions (metric tons CO2e/year)^	Estimated annual energy savings (MWh/year)^	Estimated annual renewable energy generation (MWh/year)^	Co-benefits realised^	Funding source(s)
Numeric field	Numeric field	Numeric field	Select all that apply:<br>**Economic**<br>• Job creation<br>• Revenue generation<br>• Reduced costs<br>• Increased energy security<br>• Business/technological innovation<br>• Increased labour productivity<br>• Improved labour conditions<br>• Increased economic production<br>• Reduced natural resource depletion<br>• Reduced congestion<br>• Reduced disruption of energy, transport, water and communications networks<br>**Social**<br>• Increased water security<br>• Increased food security<br>• Improved mobility and access<br>• Improved road safety<br>• Reduced fuel poverty<br>• Increased security/protection for poor/vulnerable populations<br>• Increased social inclusion, equality and justice<br>• Increased transparency and accountability<br>• Improved education and public awareness<br>• Enhanced climate change adaptation<br>• Enhanced resilience to shocks and disasters<br>**Public Health**<br>• Improved physical health<br>• Improved mental wellbeing/quality of life<br>• Improved air quality<br>• Improved preparedness for health service delivery<br>• Reduced health impacts from extreme heat or cold weather<br>• Reduced disaster/disease/contamination-related health impacts<br>• Reduced premature deaths<br>• Reduced health costs<br>**Environmental**<br>• Improved water/soil quality<br>• Improved waste management<br>• Reduced noise/light pollution<br>• Increased/improved green space<br>• Protected/improved biodiversity and ecosystem services<br>**Other impacts measured**<br>• Other impacts from climate actions<br>• Do not know	Select all that apply:<br>• Jurisdiction's own resources<br>• Regional funds and programmes<br>• National funds and programmes<br>• International (including ODA)<br>• Climate finance (carbon credits)<br>• Public-private partnerships<br>• Private partnerships (e.g., a combination of private investments)<br>• Other, please specify source(s)

Status of action in the reporting year^	Inclusion in climate action plan and/or jurisdiction development/master plan^	Total cost of action (in currency specified in 0.1)
Select from:<br>**Pre-implementation**<br>• Scoping<br>• Pre-feasibility study<br>• Feasibility finalized, but currently no finance secured<br>• Feasibility finalized, and finance partially secured<br>• Feasibility finalized, and finance fully secured<br>**Implementation**<br>• Implementation complete in the reporting year<br>• Implementation underway with completion expected in less than one year<br>• Implementation underway with completion expected in more than one year<br>**Post-implementation/Operation**<br>• Action in operation (jurisdiction-wide)<br>• Action in operation (across most of jurisdiction)<br>• Action in operation (targeted to sector/location)<br>• Other, please specify	Select from:<br>• Action is included in climate action plan and/or development/master plan<br>• Action is not included in climate action plan and/or development/master plan<br>• No climate action plan and/or development/master plan has been developed<br>• Other, please specify	

7. Univariate analysis

Univariate analysis

Before conducting the generalized linear regression model, we tested the variables status, country-income, sector, region and population alone and found evidence that actions that are in ‘Pre-operation’ are more likely to report health co-benefits (*OR* = 1.22, *p = 0.038*) compared to actions ‘In Operation’, see Table 1. We found no significant differences among regions as depicted in Table 2. Actions from high-income countries were less likely to report health co-benefits (*OR* = 0.49, *p < 0.001*), see Table 3. We found significant differences across actions from different sectors. Actions from energy (*OR* = 0.2 *p <0.001*), industry (*OR* = 0.3, *p = 0.001*) and waste (*OR* = 0.21, *p <0.001*) were significantly less likely, whereas actions from transport sector (*OR* = 1.63, *p = 0.002*) were significantly more likely to report health co-benefits compared to actions from the AFOLU sector, see Table 4.

Supplementary Table 1: *Association between reporting health co-benefits and status*

	Odds ratio	*Std. Error*	*95%-CI*	*p-value*
(Intercept: In operation)	0.42	0.06	0.32 – 0.54	<0.001
status [Pre-operation]	1.22	0.11	1.01 – 1.46	0.038
**Random Effects**				
σ^2	3.29			
$\tau_{00\ number:country}$	1.04			
$\tau_{00\ country}$	0.31			
ICC	0.29			
N_{number}	584			
$N_{country}$	71			
Observations	3835			
Marginal R^2 / Conditional R^2	0.002 / 0.293			

Supplementary Table 2: *Association between reporting health co-benefits and region*

	Odds ratio	*Std. Error*	*95%-CI*	*p-value*
(Intercept: Africa)	0.59	0.24	0.26 – 1.33	0.202
region [East Asia]	0.48	0.27	0.16 – 1.46	0.199
region [Europe]	0.99	0.45	0.41 – 2.40	0.975
region [Latin America]	0.75	0.35	0.30 – 1.88	0.537
region [Middle East]	0.29	0.24	0.05 – 1.52	0.142
region [North America]	1.29	0.69	0.45 – 3.70	0.638
region [Oceania]	0.62	0.4	0.18 – 2.17	0.452
region [South Asia]	0.85	0.64	0.20 – 3.69	0.828
region [Southeast Asia]	0.6	0.34	0.20 – 1.80	0.366
**Random Effects**				
σ^2	3.29			
$\tau_{00\ number:country}$	1.06			
$\tau_{00\ country}$	0.21			
ICC	0.28			
N_{number}	584			
$N_{country}$	71			
Observations	3835			
Marginal R^2 / Conditional R^2	0.021 / 0.294			

Supplementary Table 3: *Association between reporting health co-benefits and country-income*

	Odds ratio	*Std. Error*	*95%-CI*	*p-value*
(Intercept: HIC)	0.49	0.07	0.36 – 0.66	<0.001
LMIC	0.95	0.23	0.59 – 1.53	0.841
**Random Effects**				
σ^2	3.29			
$\tau_{00\ number:country}$	1.06			
$\tau_{00\ country}$	0.31			
ICC	0.29			
N_{number}	584			
$N_{country}$	71			
Observations	3835			
Marginal R^2 / Conditional R^2	0.000 / 0.294			

Supplementary Table 4: *Association between reporting health co-benefits and sector*

	Odds ratio	*Std. Error*	*95%-CI*	*p-value*
(Intercept: AFOLU)	1	0.18	0.71 – 1.41	1
sector [Energy]	0.2	0.03	0.15 – 0.27	<0.001
sector [Industry]	0.3	0.11	0.14 – 0.62	0.001
sector [Transportation]	1.63	0.26	1.20 – 2.22	0.002
sector [Waste]	0.21	0.04	0.14 – 0.29	<0.001
**Random Effects**				
σ^2	3.29			
$\tau_{00\ \text{number:country}}$	1.36			
$\tau_{00\ \text{country}}$	0.24			
ICC	0.33			
N_{number}	584			
N_{country}	71			
Observations	3835			
Marginal R^2 / Conditional R^2	0.162 / 0.436			

8. Analyses of main findings

Generalized-linear regression mixed effects model formula

$$Logit(Health\ co-benefit) = \beta_0 + \beta_1(Status) + \beta_2(Sector) + \beta_3(Population) + u_{country} + u_{City\ ID\ number}$$

- Logit (Health co-benefit) is the logit link function transforming the probability of the outcome (i.e. reported health co-benefit) into log-odds
- β_0 represents the intercept
- β_1, β_2, β_2 represent the fixed effect coefficients for status, sector and population
- $u_{country}$ represents the random intercept for each country
- $u_{city\ ID\ Number}$ represents the nested random intercept for each city ID number within each country

Supplementary Table 5: *Reporting of health co-benefits controlling for status, sector and population with country and city ID number as random effects*

Predictors	*Log-Odds*	*Std. Error*	*CI*	*p*
(Intercept)	-0.07	0.19	-0.44 – 0.30	0.71
status [Pre-operation]	0.1	0.1	-0.10 – 0.31	0.32
sector [Energy]	-1.62	0.16	-1.93 – -1.31	**<0.001**
sector [Industry]	-1.2	0.37	-1.93 – -0.47	**0.001**
sector [Transportation]	0.48	0.16	0.17 – 0.79	**0.002**
sector [Waste]	-1.58	0.18	-1.93 – -1.23	**<0.001**
PopScaled	0.03	0.07	-0.11 – 0.17	0.661
**Random Effects**				
σ^2	3.29			
$\tau_{00\ number:country}$	1.35			
$\tau_{00\ country}$	0.24			
ICC	0.33			
N_{number}	584			
$N_{country}$	71			
Observations	3835			
Marginal R^2 / Conditional R^2	0.163 / 0.435			

Supplementary Table 6: *Association between reporting of health co-benefits by sector and implementation status controlled for status, sector and population with country and city ID number as random effects*

Sector	Status	Odds ratio	95%-CI
Industry	Pre-operation	0.312	0.16-0.63
	In operation	0.281	0.14-0.57
Waste	Pre-operation	0.213	0.16-0.3
	In operation	0.192	0.14-0.27
Transportation	Pre-operation	1.67	1.29-2.17
	In operation	1.506	1.12-2.02
AFOLU	Pre-operation	1.034	0.73-1.46
	In operation	0.932	0.65-1.34
Energy	Pre-operation	0.204	0.16-0.27
	In operation	0.184	0.14-0.25

9. City characteristics linked to reporting a high proportion of health co-benefits

Binomial model formula

$$Logit\left(\frac{p}{1-p}\right) = \beta_0 + \beta_1(Population) + \beta_2(Region) + \beta_3(Country - income)$$

- Logit p is the link function that transforms the probability (p) of a reported health co0benefit into log-odds
- β_0 represents the model intercept
- β_1, β_2, β_3 represent the coefficients for Population, Region, and Country-income

Supplementary Table 7: *Reporting of a large proportion of health co-benefits and population, region, and country-income*

	Log-Odds
Intercept (Africa)	-0.682*<br>(0.338)
East Asia	-0.647<br>(0.374)
Europe	0.308<br>(0.34)
Latin America	0.084<br>(0.26)
Middle East	-1.065*<br>(0.493)
North America	0.382<br>(0.341)
Oceania	-0.517<br>(0.4)
South Asia	0.176<br>(0.337)
Southeast Asia	-0.294<br>(0.301)
Population	0.00000006***<br>(0.00000002)
Country-income (LMIC)	-0.08<br>(0.225)
Number of observations	584

Standard errors are reported in parentheses. *, **, *** indicates significance at 90%, 95%, and 99% level.
